# The knottin-like *Blufensin* family regulates genes involved in nuclear import and the secretory pathway in barley-powdery mildew interactions

**DOI:** 10.3389/fpls.2015.00409

**Published:** 2015-06-04

**Authors:** Weihui Xu, Yan Meng, Priyanka Surana, Greg Fuerst, Dan Nettleton, Roger P. Wise

**Affiliations:** ^1^Department of Plant Pathology and Microbiology, Center for Plant Responses to Environmental Stresses, Iowa State UniversityAmes, IA, USA; ^2^Bioinformatics and Computational Biology Graduate Program, Iowa State UniversityAmes, IA, USA; ^3^Corn Insects and Crop Genetics Research Unit, U.S. Department of Agriculture-Agricultural Research Service, Iowa State UniversityAmes, IA, USA; ^4^Department of Statistics, Iowa State UniversityAmes, IA, USA

**Keywords:** knottin, nuclear import, secretory pathway, powdery mildew, calmodulin, BSMV-VIGS, gene expression, negative regulator

## Abstract

Plants have evolved complex regulatory mechanisms to control a multi-layered defense response to microbial attack. Both temporal and spatial gene expression are tightly regulated in response to pathogen ingress, modulating both positive and negative control of defense. BLUFENSINs, small knottin-like peptides in barley, wheat, and rice, are highly induced by attack from fungal pathogens, in particular, the obligate biotrophic fungus, *Blumeria graminis* f. sp. *hordei (Bgh)*, causal agent of barley powdery mildew. Previous research indicated that *Blufensin1* (*Bln1*) functions as a negative regulator of basal defense mechanisms. In the current report, we show that BLN1 and BLN2 can both be secreted to the apoplast and *Barley stripe mosaic virus* (*BSMV*)-mediated overexpression of *Bln2* increases susceptibility of barley to *Bgh*. Bimolecular fluorescence complementation (BiFC) assays signify that BLN1 and BLN2 can interact with each other, and with calmodulin. We then used *BSMV*-induced gene silencing to knock down *Bln1*, followed by Barley1 GeneChip transcriptome analysis, to identify additional host genes influenced by *Bln1*. Analysis of differential expression revealed a gene set enriched for those encoding proteins annotated to nuclear import and the secretory pathway, particularly Importin α1-b and Sec61 γ subunits. Further functional analysis of these two affected genes showed that when silenced, they also reduced susceptibility to *Bgh*. Taken together, we postulate that *Bln1* is co-opted by *Bgh* to facilitate transport of disease-related host proteins or effectors, influencing the establishment of *Bgh* compatibility on its barley host.

## Introduction

Obligate fungal biotrophs, i.e., pathogens that require their host to survive, are a major threat to crop production worldwide. To establish biotrophy, the fungus must penetrate cell walls, suppress defense, and establish haustoria for nutrient acquisition (Dodds et al., 2004; Micali et al., 2011; Mentlak et al., 2012). In general, these pathogens interfere with recognition at the host plasma membrane or secrete effector proteins, often through feeding structures termed haustoria, into the plant cell cytosol that alter resistance signaling or the downstream manifestation of resistance responses. Many cloned effectors are small proteins of unknown function containing a signal for secretion into the apoplast; how these effectors gain entry into host cells and contribute to pathogen colonization has been a major focus to understand the underlying mechanisms determining pathogenicity (Rovenich et al., 2014; Stotz et al., 2014).

The host responds with an integrated multi-layer defense system. Typically, pathogen-associated molecular patterns (PAMPs) trigger the initial activation of non-specific, innate immune responses, currently termed PAMP Triggered Immunity (PTI) (Macho and Zipfel, 2014). These include the transcription of thousands of stress-related genes, as well as production of antimicrobial metabolites and peptides during early stages of pathogen invasion. A second layer, designated Effector-Triggered Immunity (ETI) generally follows gene-for-gene interactions, in which specific resistance (R) proteins initiate a signal cascade when they recognize, either directly or indirectly, corresponding effectors delivered by the pathogen (Bent and Mackey, 2007; Jacob et al., 2013; Cesari et al., 2014).

Host factors that are activated and recruited by pathogen effectors interfere with different layers of the plant defense response. These plant factors are either called negative regulators of plant defense or susceptibilty factors, which are co-opted by the pathogen to optimize growth and parasitism; both are encoded by susceptibility (*S*) genes (Vogel et al., 2002, 2004; Hückelhoven et al., 2013; Lapin and Van Den Ackerveken, 2013; Van Schie and Takken, 2014). Mutation of an *S* gene has the potential to alter the plants susceptibility and lead to resistance, an important feature that is often used in breeding. For example, the cell wall has long been recognized as a major barrier against pathogen infection (Bellincampi et al., 2014; Malinovsky et al., 2014). PMR5 and PMR6 are two potential susceptibility factors identified in Arabidopsis. Mutations in *pmr5* [defective in a gene encoding a predicted endoplasmic reticulum (ER) protein] and *pmr6* (defective in a cell wall-degrading pectate lyase-like gene) genes both affect pectin composition of the cell wall, thus increasing Arabidopsis resistance to powdery mildew (Vogel et al., 2002, 2004). Other examples include *xa5*, encoding a subunit of transcription factor IIA (Iyer and McCouch, 2004; Jiang et al., 2006), and *xa13*, encoding a plasma membrane protein and essential for pollen development (Chu et al., 2006). Both loss-of-function mutants to bacterial blight have been used successfully in rice cultivation (Iyer-Pascuzzi and McCouch, 2007).

A classic case in barley is the well-characterized *Mlo* gene that encodes a transmembrane protein, which negatively regulates penetration resistance to powdery mildew (Büschges et al., 1997). Loss of function *mlo* mutants result in durable and broad-spectrum resistance, which has been wildly adapted for cultivation in Europe (Büschges et al., 1997; Panstruga, 2005; Acevedo-Garcia et al., 2014). MLO2, an *Arabidopsis thaliana* homolog of the barley *S*-gene *Mlo*, was found to be the target of the *Pseudomonas syringae* effector HopZ2 (Lewis et al., 2012).

BAX INHIBITOR-1 (BI-1) inhibits BAX-induced PCD in yeast and Arabidopsis; additionally, BI-1 modulates cell-wall-associated defense and contributes to establishing full compatibility of barley with the obligate biotrophic fungus, *Blumeria graminis* f. sp. *hordei* (*Bgh*), causal agent of powdery mildew disease (Eichmann et al., 2010). Interestingly, overexpression of BI-1 was found to negatively regulate penetration resistance mediated by *mlo* and almost restored the penetration efficiency (PE) of *Bgh* to wild-type levels (Hückelhoven et al., 2003), suggesting these genes have important roles in a complex interconnected network. The MLO protein in barley negatively regulates the actin-dependent resistance pathway, and the actin cytoskeleton is thought to contribute to the establishment of effective barriers at the cell periphery against fungal access (Miklis et al., 2007). The RAC/ROP family G-protein RACB, another potential host susceptibility factor, is also involved in the modulation of actin reorganization and cell polarity in the interaction of barley with *Bgh* (Opalski et al., 2005).

We previously reported the discovery of the monocot-specific Blufensin family of cysteine-rich, peptides, which negatively impact plant defense (Meng et al., 2009). The *Bln1* and *Bln2* transcripts are highly upregulated in response to infection by a wide array of fungal pathogens, including *Blumeria, Puccinia, Cochliobolus*, and *Fusarium* spp., as compared to uninfected control plants. The genes that encode these peptides are so far unique to the cereal grain crops barley, wheat, and rice, and the resulting proteins are similar to knottins, a diverse family of proteins characterized by a unique disulfide through disulfide knot (Gracy et al., 2008).

In the work described herein, we used BLN-GFP fusion constructs to demonstrate that BLN1 and BLN2 can be secreted into the apoplast. Bimolecular fluorescence complementation (BiFC) assays (Kerppola, 2006) suggest that BLN1 and BLN2 interact with calmodulin, as well as each other. *Barley stripe mosaic virus* (*BSMV)*-mediated *Bln* overexpression increased susceptibility of barley to *Bgh*. BSMV–Virus Induced Gene Silencing (VIGS) coupled with a Barley1 GeneChip transcriptome analysis, identified additional genes in the *Blufensin1* (*Bln1*) network. These candidates appear to have key roles in *R*-gene mediated and innate immunity networks, thus, the functional identification of their precise roles will be a significant step in understanding plant defense.

## Results

### BLN1 and BLN2 can be secreted into the apoplast

In previous research, BSMV-VIGS of *Bln1* decreased barley susceptibility to *Bgh* in compatible interactions. Likewise, single cell transient overexpression of *Bln1* significantly increased accessibility toward virulent *Bgh*. Moreover, silencing of *Bln1* in plants harboring the *Mildew locus o* (*Mlo)* susceptibility factor decreased accessibility to *Bgh*, suggesting BLN1 functions in parallel with or upstream of MLO to modulate penetration resistance (Meng et al., 2009).

**Figure 1:**
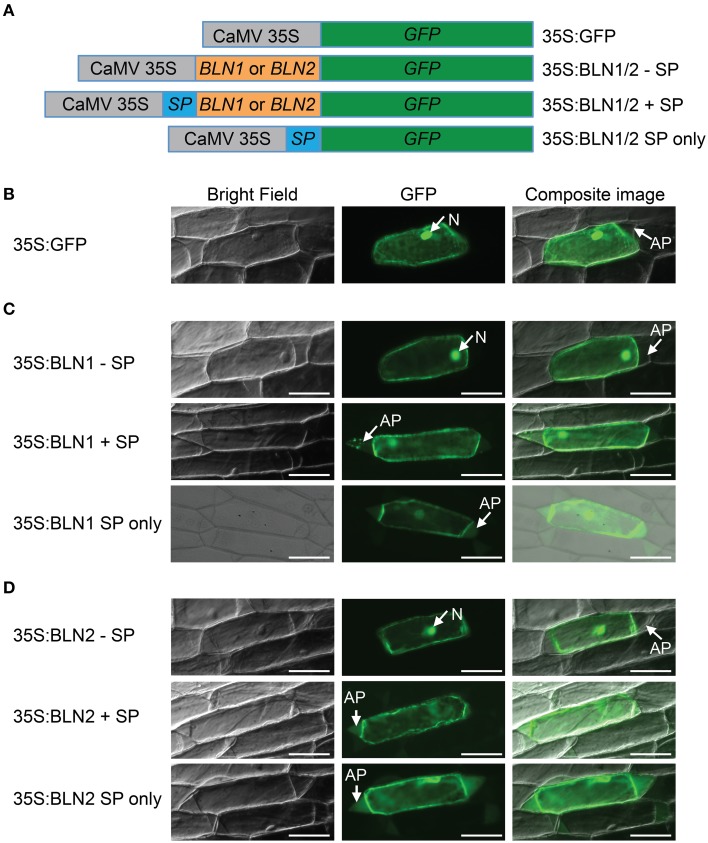
**Subcellular localization of BLN1 and BLN2**. **(A)** Schematic diagram of expression constructs. Gray boxes represent *Cauliflower Mosaic Virus* (CaMV) 35S promoter; the green, orange, and the blue boxes represent coding regions for GFP, mature BLN1/BLN2, and BLN1/BLN2 signal peptides, respectively. The CaMV 35S promoter was used to drive gene expression. The GFP coding sequence is fused to *Bln1* and *Bln2* without signal peptide-coding region (35S:BLN1/2—SP), to *Bln1* and *Bln2* with signal peptide-coding region (35S:BLN1/2 + SP), and to BLN1/2 signal peptide-coding region only (35S:BLN1/2 SP only). The construct harboring GFP coding sequence alone was used as a subcellular localization control. **(B)** Microscopic observation of GFP signal in onion epidermal cells after plasmolysis. GFP signal was observed in the cytoplasm and nucleus region in cells expressing GFP alone. No GFP signal was observed in apoplast region. **(C,D)** Microscopic observation of GFP signal in onion epidermal cells after plasmolysis. GFP signal was observed in apoplast when GFP was fused to full-length BLN1 or BLN2 with signal peptide (35S:BLN1 + SP, 35S:BLN2 + SP), as well as in cells expressing GFP fused to signal peptides from BLN1 and BLN2 (35S:BLN1 SP only, 35S:BLN2 SP only). By contrast, no GFP signal was observed in the apoplastic region in cells expressing GFP fused to BLN1 or BLN2 without signal peptides from BLN1 and BLN2 (35S:BLN1-SP, 35S:BLN2-SP). Left column: bright field images; middle column: fluorescence microscopic images of GFP; right column: composite images of the GFP and bright light images. AP, apoplast; N, Nucleus. Bar = 100 μm.

Computational analysis of the BLN1 and BLN2 signal peptides (SP) predicted that BLN could be secreted into the apoplast, and thus, may act as ligands to generate a signal transduction cascade, influencing *Bgh* accessibility (Meng et al., 2009). To test this hypothesis, six different *Bln*-*GFP* fusion constructs were assembled for bombardment into onion epidermal cells, [BLN1 or 2 minus SP (35S:BLN1/2–SP), BLN1 or 2 plus SP (35S:BLN1/2 + SP), and BLN1 or 2 SP only (35S:BLN1/2 SP only)] (Figure 1A).

Because GFP is unstable at low pH, to visualize its expression in the apoplast, onion epidermal cells were treated with 20 mM Pipes-KOH (pH 7.0) to neutralize the pH according to Genovesi et al. (2008) (see Materials and Methods). The pH 7.0 medium neutralizes the normally acidic apoplast, facilitating the visualization of GFP-mediated fluorescence. As illustrated in Figures 1C,D (middle panel), GFP fluorescence was detected in the apoplast, cytoplasm and nuclei of plasmolysed cells when transformed with the full-length *Bln1* or *Bln2* ORFs fused with GFP. Similar results were obtained when constructs harboring *GFP* fused with coding sequences for signal peptides from BLN1 or BLN2 (Figures 1C,D, lower panel). By contrast, GFP fluorescence was found only in the cytoplasm or the nucleus when onion epidermal cells were bombarded with constructs absent the signal peptides (Figures 1C,D, upper panel), similar to the GFP-only control (Figure 1B); no visible fluorescent signal was observed in the apoplastic region. The above results indicate that the BLN1 and BLN2 signal peptides can direct protein secretion, and both BLN1 and BLN2 can be secreted from the cytoplasm into the apoplast.

### BSMV-VOX: a new *BSMV*-mediated overexpression system for functional analysis of *Bln1* and *Bln2*

**Figure 2:**
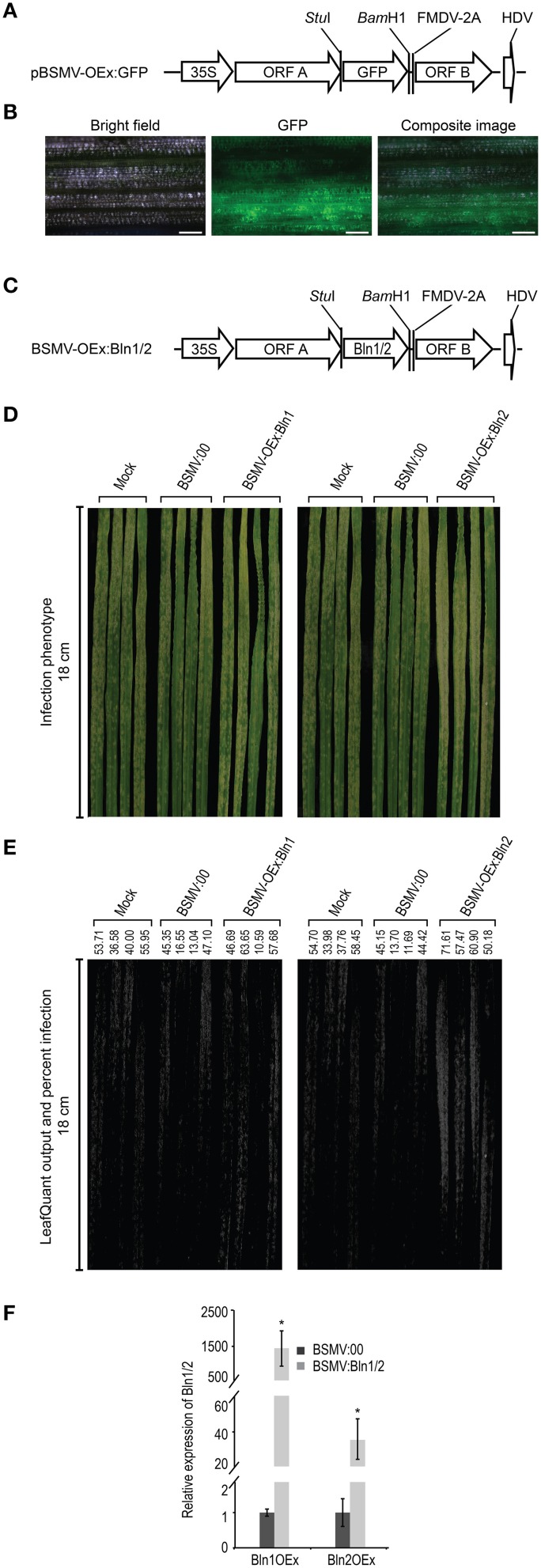
***BSMV*****-mediated over-expression of** ***Bln1/2***. **(A)** Schematic diagram of *BSMV*-mediated GFP over-expression (VOX) construct. The coding sequence for the fusion protein of GFP and foot- and-mouth disease virus (*FMDV*)-2A self-cleavage peptide was digested by *Bgl*II and *Kpn*I, ligated into the construct BSMV: γ, which harbors the BSMV γ-subgenome. Subsequent digestion with *Bgl*II and *Kpn*I, resulted in *BSMV*-mediated GFP over-expression construct pBSMV-OEx:GFP. **(B)** Microscopic observation of GFP signal (middle column) in barley leaves (black hull-less) co-bombarded with *BSMV*-mediated GFP over-expression construct pBSMV-OEx:GFP, BSMV:α, and BSMV:β. The efficacy of the observed GFP signal is 100% in leaves that showed *BSMV* infection symptom. Bar = 100 μm. **(C)** Schematic diagram of *BSMV*-mediated BLN1/2 over-expression construct pBSMV-OEx:Bln1/2. PCR amplified fragments for Bln1/2 coding regions were digested by *Stu*I and *Bam*H1 and inserted into *Stu*I and *Bam*H1 digested pBSMV-OEx:GFP, generating constructs pBSMV-OEx:Bln1 and pBSMV-OEx:Bln2. **(D)** Phenotype of *Bgh* infected leaves treated with buffer (Mock), BSMV:00 control, and pBSMV-OEx:Bln1 (*Bln1* over-expression) (left column); buffer (Mock), BSMV:00 control, and *Bln2* over-expression (pBSMV-OEx:Bln2) (right column). **(E)** LeafQuant infection phenotype images and quantification of *Bgh* hyphal growth on leaves treated with buffer (mock), BSMV:00 control, and over-expression for *Bln1* and *Bln2* (See Table 1). **(F)** Quantitative RT-PCR analyses for *Bln1* and *Bln2* levels in leaves treated by BSMV-mediated over-expression Bln1OEx and BlnOEx. Bars represent standard error calculated from at least four independent plants for each treatment from two replicate experiments shown in this figure. The average *Bln1* and *Bln2* levels in BSMV:00 control were set to 1.00 (^*^ designates that *p* < 0.05).

As described above, we developed a bombardment based BSMV-VIGS system for high-throughput silencing of candidate genes involved in interactions with the barley powdery mildew fungus (Meng et al., 2009). To complement these gene-silencing studies, we further developed *BSMV* as a transient overexpression system (BSMV-VOX) for functional analysis in both host and pathogen. To generate the expected cleavage products from the artificial fusion proteins, a 54-nucleotide sequence encoding the 18 amino-acid foot and mouth virus peptide (FMDV-2A) was inserted in front of the 5′ end of BSMV:γ ORF B. The ORF encoding GFP was inserted between the *Stu*I and *Bam*H1 sites before FMDV 2A as a visible marker to monitor overexpression (Figure 2A). These GFP and BSMV:γB coding regions are fused in-frame via the FMDV 2A coding sequence. The FMDV 2A peptide mediates the primary *cis*-“cleavage” of the FMDV polyprotein in a cascade of processing events that ultimately generate the mature FMDV proteins. Subsequently, FMDV 2A efficiently generates the expected cleavage products from the artificial fusion proteins in cells (Furler et al., 2001). The *BSMV*-mediated overexpression construct (pBSMV-OEx) was then co-bombarded with BSMV:α and BSMV:β to barley cultivar Black Hulless, which is susceptible to *BSMV*. Overexpression of *GFP* (pBSMV-OEx:GFP) was used to examine the efficacy of overexpression with this approach. Microscopic observation showed that all leaves with *BSMV* infected stripe and mosaic symptoms also exhibit green fluorescence as detected by UV microscopy (Figure 2B), signifying the robustness of BSMV-VOX system for transient gene overexpression. In addition, due to the systemic infection of *BSMV*, the BSMV-VOX system results in transient gene overexpression throughout *BSMV*-infected barley leaves (Lee et al., 2012), as compared to single-cell-overexpression in epidermal cells (Meng et al., 2009). Therefore, the infection phenotypes can be observed by the naked eye, hyphal growth and associated symptoms can be quantified digitally, and the target gene expression levels can be assayed by quantitative real-time reverse transcriptase PCR (qRT-PCR), in the absence of stable transformation.

### Overexpression of *Bln2* increases susceptibility in compatible interactions

Our newly developed BSMV-VOX system (described above) was adopted to further corroborate the function of *Bln* genes in barley immunity to *Bgh*. Full-length *Bln1-1* and *Bln2* ORFs were substituted in place of *GFP* in the BSMV-VOX vector, pBSMV-OEx:GFP, to create the expression constructs BSMV-OEx:Bln1 and BSMV-OEx:Bln2 (Figure 2C). pBSMV-OEx:Bln1 or pBSMV-OEx:Bln2 plasmids were then co-bombarded with the BSMV:α and BSMV:β separately into 7 day old Black Hulless seedlings. After 7 days, sap from *BSMV* infected barley leaves was used to mechanically inoculate barley cultivar HOR11358 (*Mla9*). Twelve days after overexpression, plants were subsequently challenged with the virulent *Bgh* isolate 5874 (*avr_a_*_9_). Control bombardments were performed with the BSMV:00 construct (see Materials and Methods). Systemic overexpression of *Bln2* in whole barley leaves significantly increased susceptibility in compatible barley-*Bgh* interactions (*p* = 0.0194). Although one can observe a small increase in *Bgh* colony proliferation on *Bln1*-OEx barley leaves, this did not result in a significant difference in quantifiable growth (Figures 2D,E, Table 1). This contrasts with our previous result using transient single-cell-overexpression in barley epidermal cells (Meng et al., 2009), and may be due to the differential resistance of barley genotypes to *BSMV* (Hein et al., 2005), which could further influence the phenotypic effects of *BSMV*-mediated overexpression, as opposed to single cell bombardment assays (which contain no *BSMV*).

**Table 1:** **Linear model analysis of BSMV induced gene overexpression and silencing on** ***Bgh*** **infection^a^**.

**Treatment^b^**	**Control**	**Percent infection^c^**		**Standard error^d^**	***T*-value**	**Adjusted *p*-value^e^**
		**Treatment**	**Control**			
***Bln1***						
BSMV:00	Mock	30.510	46.560	12.850	−1.249	0.3890
12219 p1 OEx	BSMV:00	44.653	30.510	12.850	1.101	0.4680
***Bln2***						
BSMV:00	Mock	28.780	46.223	9.756	−1.792	0.1812
26496 p1 OEx	BSMV:00	60.040	28.780	9.756	3.208	0.0194
***Imp α-1b***						
3615 p1	BSMV:00	30.401	65.812	6.625	−5.345	0.0007
***Sec61 γ***^f^						
3680 p2	BSMV:00	35.771	77.311	8.751	−4.747	0.0020

a *A linear model analysis was performed relative to BSMV with empty vector using the MULTCOMP package in R*.

b *Mock and BSMV with indicated silencing or overexpression plasmids were compared against BSMV with empty vector; OEx, overexpression*.

c *LeafQuant-VIGS distinguishes white Bgh hyphae from dark green leaves as a quantitative measure of Bgh associated hyphal growth (see Materials and Methods). LeafQuant-VIGS converts the images to gray scale and outputs histograms of the hyphal distribution per leaf, which then reports mean, median, and quantiles of the results as a csv (comma separated values) file for further processing (Whigham et al., 2015). The average of percent infection for each treatment across replicates is shown here*.

d *Standard error is the estimate of how far the sample mean is likely to be from the population mean*.

e *p-values were adjusted for multiple testing using the Dunnett method (Dunnett, 1955). Treatments with adjusted p ≤ 0.05 were considered significantly different from the control*.

f *The results for mock samples compared to empty vector were not significant for Sec61*.

Transcript accumulation of *Bln1* and *Bln2* was assayed to monitor the level of gene overexpression. Third leaves of BSMV-treated plants were used for qRT-PCR assays at 24 HAI with *Bgh*. Barley *Actin* mRNA was used as an internal quantitative control for all samples. Results of qRT-PCR demonstrated the distinct induction of *Bln1* and *Bln2* transcripts in *Bgh* inoculated leaves that harbored overexpression constructs as compared to BSMV:00 inoculated plants (Figure 2F).

### Interaction of BLN1 and BLN2

**Figure 3:**
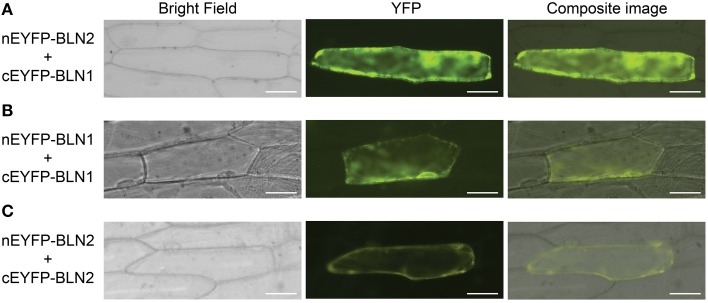
**Bimolecular fluorescence complementation (BiFC) assay for BLN1 and BLN2 interactions**. The *Bln1* and *Bln2* full length ORFs were PCR-amplified and cloned into *Eco*RI-*Bam*HI sites of pSAT4-nEYFP-C1 or pSAT4-cEYFP-C1 to generate pSAT4-nEYFP-Bln1, pSAT4-nEYFP-Bln2, pSAT4-cEYFP-Bln1 pSAT4-cEYFP-Bln2 respectively. The designated plasmid combinations were co-bombarded into onion epidermal cells. The bright field and fluorescent images were taken using the Zeiss Axiovert 100 microscope equipped with the appropriate YFP filter. Statistical analysis of YFP count data is presented in Table 2. **(A)** Microscopic observation for interaction between BLN1 and BLN2. **(B)** Microscopic observation for BLN1 self-interaction. **(C)** Microscopic observation for BLN2 self-interaction.

Next, we were interested to see if the BLN1 and BLN2 secreted small peptides could physically interact as BLN complexes to facilitate cellular signaling. To test this hypothesis, bimolecular fluorescent complementation (BiFC) assays (Kerppola, 2006) were performed to test the interaction between BLN1 and BLN2. *Bln1* and *Bln2* full-length open reading frames were fused to both N-terminal and C-terminal halves of yellow fluorescent protein (YFP), respectively, and co-expressed in onion epidermal cells. As shown in Figure 3A and Table 2, the interaction of N-terminal BLN2 and C-terminal BLN1 re-comprised YFP activity. We did not observe YFP fluorescence in tests with the reciprocal (N-terminal BLN1 and C-terminal BLN2) constructs, implying some conformational constraints on successful interactions (Table 2). This may be due to the orientation of the GFP tag in relation to the interacting interface. This non-reciprocity was also observed in BiFC interaction experiments among *Bgh* effector proteins and barley small heat shock proteins (Ahmed et al., 2015).

**Table 2:** **Mixed linear analysis of mutations in the BLN1 and BLN2 IQ domain and cysteines and their effect on forming heteroduplexes^a^**.

**Treatment**	**Mean YFP cell counts^b^**	**Estimate^c^**	***T*-value**	**Adjusted *P*-value^d^**
BLN1 and BLN2^e^	0.00	–	–	–
BLN1-C36G and BLN2	0.33	−0.33	−0.38	0.9855
BLN1-C45G and BLN2	1.00	−1.00	−1.13	0.6421
BLN1-Q30G and BLN2	2.33	−2.33	−2.63	0.0891
BLN1-Q42G and BLN2	0.67	−0.67	−0.75	0.8632
BLN2 and BLN1	8.00	–	–	–
BLN2-C37G and BLN1	1.00	7.00	5.63	0.0016
BLN2-C47G and BLN1	0.00	8.00	6.44	0.0007
BLN2-Q30G and BLN1	0.33	7.67	6.17	0.0009
BLN2-Q44G and BLN1	2.00	6.00	4.83	0.0042

a *A mixed linear model analysis was done using PROC MIXED of the SAS Software. Contrasts were designed to test the differences between the control and the treatment with cell counts as the response*.

b *Represents mean of total cells exhibiting YFP from three independent biological replications. The appearance of YFP fluorescing cells was equivalent among mutant or wild-type bombarded constructs*.

c *Difference between least square means for the total YFP cells in control vs. the treatment*.

d *P-values were adjusted for multiple testing using the methods of Dunnett (1980) and Hsu (1992). Treatments with adjusted p ≤ 0.05 were considered significantly different from the BLN1 and BLN2 wild-type control interactions*.

e *These BLN1 and BLN2 reciprocal constructs serve as internal negative controls for non-specific (background) interactions*.

DISULFIND software (Ceroni et al., 2006) predicted that the cysteines in BLN1 and BLN2 form disulfide bonds; these are expected to stabilize knottin protein structures, which may be critical for interactions with other proteins (Combelles et al., 2008; Gracy et al., 2008). To test if these two conserved cysteines may be involved in the interaction interface between BLN1 and BLN2 (Kerppola, 2006), Cys36 and Cys45 in BLN1 and Cys37 and Cys47 in BLN2 were mutated to Gly and the resulting BiFC constructs were co-bombarded into onion epidermal cells. As shown in Table 2, the average number of observed fluorescent cells from three independent replications was significantly reduced (adjusted *p* < 0.0016). The reciprocal construct described above, as well as each of the BLN1 and BLN2 site-directed mutants also serve as negative controls for non-specific interactions (Kerppola, 2006). Interestingly, co-bombardment of constructs harboring BLN1 fused to the N-terminal and C-terminal halves of YFP also showed YFP activity (Figure 3B); similar results were also observed for BLN2 (Figure 3C). These results suggest that BLN family members can not only interact with each other, but also dimerize or polymerize with themselves. Even so, mutations in conserved residues may compromise protein stability, thus, this preliminary result should be viewed with caution without direct evidence that the mutant proteins actually accumulate.

### Interactions between BLN family members and calmodulin

**Figure 4:**
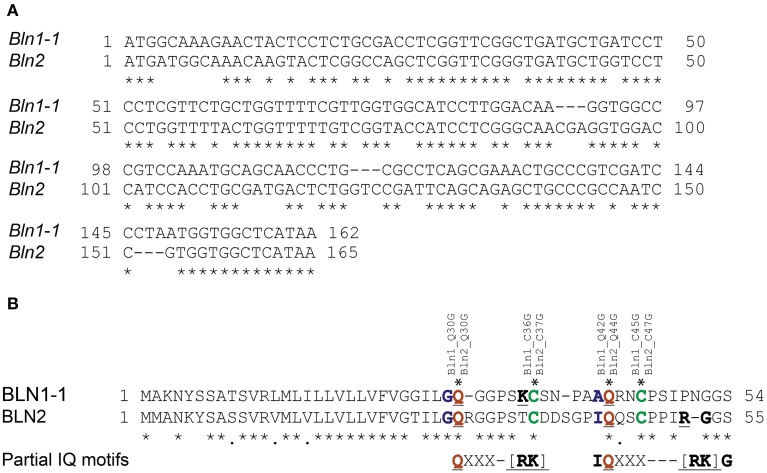
**Alignment of** ***Bln1*** **and** ***Bln2*** **with the positions of conserved cysteines and potential partial IQ motifs. (A)** Nucleotide alignment of *Bln1* and *Bln2* open reading frames. A “^*^” indicates conserved nucleotides. **(B)** Amino acid alignment of BLN1 and BLN2. The conserved glutamines (Q) in the partial IQ motifs are shown in red (with blue adjacent), cysteines (C) are shown in green. The conserved amino acids in potential partial IQ motifs (compared to the complete consensus [FILV]Qxxx[RK]Gxxx[RK]) are underlined. For BLN1, Q to G mutations were inserted at amino acid residues 30 and 42, and C to G mutations at residues 36 and 45. For BLN2, Q to G mutations were inserted at amino acid residues 30 and 44, and C to G mutations at residues 37 and 47.

**Figure 5:**
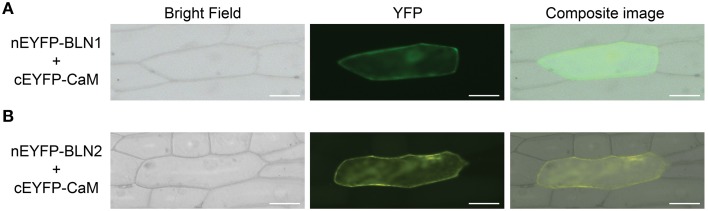
**Bimolecular fluorescence complementation (BiFC) assay for interaction between BLN1/2 and CaM**. The *Bln1* and *Bln2* full length ORFs were PCR-amplified and cloned into *Eco*RI-*Bam*HI sites of *Eco*RI-*Bam*HI sites of pSAT4-nEYFP-C1 or pSAT4-cEYFP-C1 to generate pSAT4-nEYFP-Bln1, pSAT4-nEYFP-Bln2, pSAT4-cEYFP-CaM respectively. The designated plasmid combinations were co-bombarded into onion epidermal cells. The bright-field and fluorescent images were taken using the Zeiss Axiovert 100 microscope equipped with appropriate YFP filter. Statistical analysis of YFP count data is presented in Table 3. **(A)** Microscopic observation for interaction between BLN1 and CaM. **(B)** Microscopic observation for interaction between BLN2 and CaM.

Calmodulin (CaM) plays a pivotal role in controlling an abundance of Ca^2+^-based cellular signaling events (Berridge et al., 2003) and functions in response to changes in cellular calcium levels by interacting with various targets, including those in plant immunity (Yamniuk and Vogel, 2004; Du et al., 2009). These targets include IQ (isoleucine-glutamine; consensus sequence = [FILV]Qxxx[RK]Gxxx[RK])- (Rhoads and Friedberg, 1997; Bahler and Rhoads, 2002) and partial-IQ-motif containing proteins (Houdusse and Cohen, 1995; Munshi et al., 1996; Sienaert et al., 2002). As shown in Figure 4, sequence alignments indicate that BLN family members contain partial IQ motifs. Moreover, previous results from silencing and overexpression experiments suggested that BLN family members possess an *S*-gene function somewhat similar to MLO, a calmodulin (CaM)-binding protein in plant defense (Kim et al., 2002). Thus, we were interested to investigate if BLN1 or BLN2 could physically interact with CaM. Both *Bln1* and *Bln2* full-length open reading frames were fused to the N-terminal half of YFP, *CaM* was fused to C-terminal half, and co-expressed in onion epidermal cells. As illustrated in Figures 5A,B and Table 3, significant YFP fluorescence was observed, indicating a possible interaction of BLN1/BLN2 with CaM. To further examine the function of glutamine residues in the BLN1 and BLN2 partial IQ motifs, Gln30 and Gln42 in BLN1, and Gln30 and Gln44 in BLN2 were mutated to Gly. Quantification for fluorescent cells indicated that these mutations significantly reduced the numbers of observed interactions between BLN and CaM (adjusted *p* < 5.43^E-04^), suggesting that these two glutamine residues in BLN1 and BLN2 are necessary to facilitate the full-strength interaction with CaM (Table 3). Interestingly, the Gln mutations in BLN2 also negatively impacted BLN1-BLN2 interactions (Table 2, adjusted *p* < 0.0042).

**Table 3:** **Mixed linear analysis of mutations in the BLN1 and BLN2 IQ domain and cysteines and their effect on binding with CaM^a^**.

**Treatment**	**Mean YFP cell counts^b^**	**Estimate^c^**	***T*-value**	**Adjusted *P*-value^d^**
BLN1 and CaM	57.00	–	–	–
BLN1-C36G and CaM	2.33	54.67	17.28	4.37E-07
BLN1-C45G and CaM	0.33	56.67	17.91	3.30E-07
BLN1-Q30G and CaM	8.33	48.67	15.38	1.08E-06
BLN1-Q42G and CaM	2.67	54.33	17.17	4.59E-07
BLN2 and CaM	32.33	–	–	–
BLN2-C37G and CaM	1.00	31.33	6.13	3.85E-04
BLN2-C47G and CaM	1.33	31.00	6.06	4.19E-04
BLN2-Q30G and CaM	0.00	32.33	6.32	2.99E-04
BLN2-Q44G and CaM	2.33	30.00	5.87	5.43E-04

a *A mixed linear model analysis was done using PROC MIXED of the SAS Software. Contrasts were designed to test the differences between the control and the treatment with cell counts as the response*.

b *Represents mean of total cells exhibiting YFP from three independent biological replications. The appearance of YFP fluorescing cells were equivalent among mutant or wild-type bombarded constructs*.

c *Difference between the least square means for the total YFP cells in control vs. the treatment*.

d *P-values were adjusted for multiple testing the methods of Dunnett (1980) and Hsu (1992). Treatments with adjusted p ≤ 0.05 were considered significantly different from the BLN1, BLN2, and CaM wild-type control interactions*.

Both BLN1 and BLN2 are cysteine-rich small peptides (Meng et al., 2009). These cysteines are positioned in or close to the partial IQ motifs (Figure 4). It is predicted that these cysteines may form inter- or intra-molecular disulfide bonds to maintain a structure supporting protein-protein interactions and cysteines in CaM targets are important for CaM-target interactions (Moore et al., 1999). To investigate the possible function of these two cysteines in the interaction between BLN and CaM, the Cys to Gly site-directed mutants described above were used in pairings with CaM, resulting in significantly reduced fluorescence activity (adjusted *p* < 4.19^E-04^) (Table 3). These data indicate that these cysteines play a role, either directly or indirectly, in the interaction between BLN protein and CaM.

### Identification of *Bln*-mediated response pathways

The data presented above, combined with previous functional studies (Meng et al., 2009), indicate that the monocot-specific, BLN small secreted peptides negatively regulate barley-*Bgh* interactions. To identify genes influenced by *Bln1* function, we took a mutational approach and used the BSMV-VIGS system to knock down *Bln1* (Contig12219_at). We then performed Barley1 GeneChip expression profiling on the silenced plants to discover additional genes that impact *Bln1*-mediated regulation of immunity.

**Figure 6:**
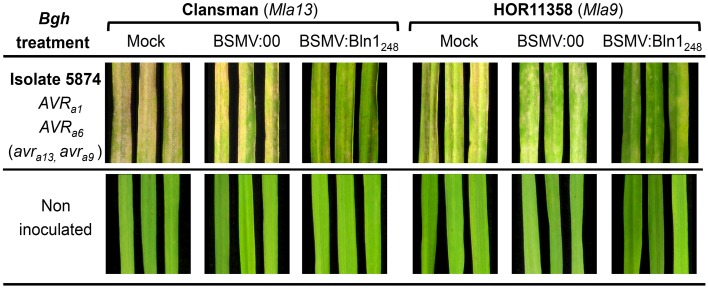
**Barley1 GeneChip expression profiling design and phenotypes upon** ***Bgh*** **infection**. Transcript profiling was based a split-plot design with 5 replications as blocks, *Bgh* treatment as the whole-plot factor, and all combinations of genotype [cv. Clansman (*Mla13*) and cv. HOR11358 (*Mla9*)] and VIGS treatment [Buffer control (mock), BSMV:00 (empty vector), and BSMV:Bln1_248_] as the split-plot factor for a total of 60 Barley1 GeneChip hybridizations. Seven-day-old plants were treated with Mock, BSMV:00, and BSMV:Bln1_248_. Twelve days after buffer and *BSMV* treatments, plants were inoculated with the compatible *Bgh* isolate 5874 (*AVR_a_*_1_, *AVR_a_*_6_, *avr_a_*_9_, *avr_a_*_13_), or non-inoculated. Leaves were harvested at 32 h after *Bgh* inoculation. Five leaves for each treatment were used for phenotyping 7 days after *Bgh* inoculation.

Figure 6 illustrates the basic matrix of the experiment. Key contrasts were designed to compare differences in transcript accumulation among *Bln1*-silenced plants relative to BSMV:00 (empty vector) controls. Comparison of BSMV:00 to mock (buffer-treated) controls enabled us to detect possible confounding effects of BSMV. To account for background-specific differences, we utilized two host genotypes; both compatible with our *Bgh* 5874 isolate and previously demonstrated to be good hosts for BSMV-VIGS experiments (Hein et al., 2005; Meng et al., 2009; Meng and Wise, 2012).

We performed five independent biological replications of a split-plot experimental design (shown in Figure 6) with replications as blocks, *Bgh* treatment as the whole-plot factor, and all combinations of genotype [Clansman (*Mla13*), HOR11358 (*Mla9*)] and VIGS treatment [Buffer control (mock), BSMV:00 (empty vector), BSMV:Bln1_248_] as the split-plot factor for a total of 60 GeneChip hybridizations. Ten seedlings were used as a split-plot experimental unit. Twelve days after VIGS treatment, plants were transferred to a growth chamber where half of the plants in each replication were challenged with the compatible *Bgh* isolate 5874; the other half remained un-inoculated. At 32 h after inoculation (HAI), 5 of the 10 leaves from each treatment were harvested for RNA isolation; this timepoint has the highest differential *Bln1* transcript accumulation in prior experiments (Meng et al., 2009), and is after initial establishment of the perihaustorial interface (Caldo et al., 2004). The remaining 5 leaves were used to document infection phenotypes 7 days after inoculation (representative experiments shown in Figure 6).

**Figure 7:**
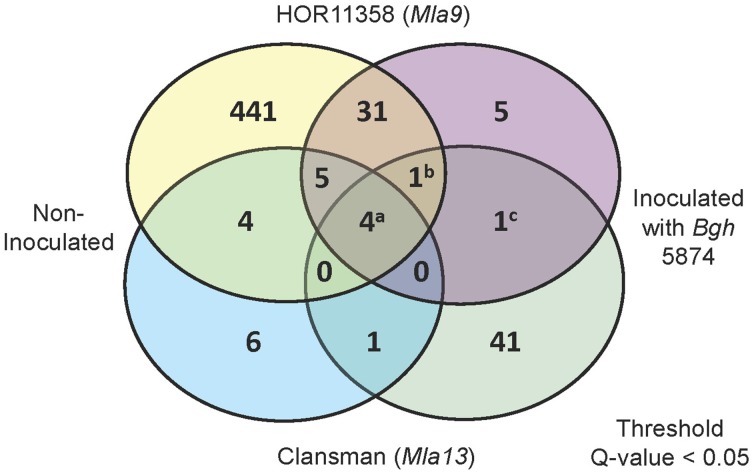
**Summary of differentially expressed genes in** ***Bln1*****-silenced leaves as compared to BSMV:00 controls**. Venn diagram represents differentially expressed genes (Threshold: *q* < 0.05) with and without inoculation with *Bgh* for both HOR11358 (*Mla9*; upper circles) and Clansman (*Mla13*; lower circles) background plants. Contrasts were assessed between empty vector (BSMV:00) and *Bln1* silenced (BSMV:Bln1_248_) plants. Superscripts (a) Contig12608_at, HS16M03u_x_at, HV10C01u_s_at, Contig3615_at (*Importin α-1b*); (b) Contig12219_at (*Bln1*); (c) Contig3680_s_at (*Sec61 γ*).

**Figure 8:**
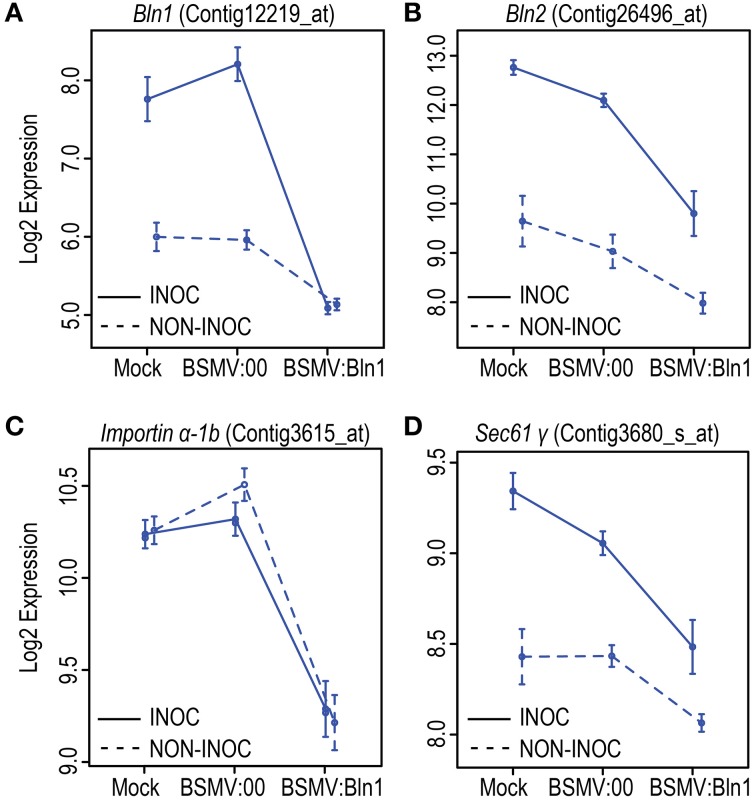
**Log2 expression level results from Barley1 GeneChip profiling of** ***Bln1**, **Bln2**, **Importin*** **α-1b, and** ***Sec61*** **γ in** ***Bln1*****-silenced leaves**. *Barley stripe mosaic virus* (*BSMV*)-Virus Induced Gene silencing was performed on HOR11358 (*Mla9*) plants as described in Figure 6. Twelve days after buffer and *BSMV* treatments, plants were inoculated with the compatible *Bgh* isolate 5874 (*AVR_a_*_1_, *AVR_a_*_6_, *avr_a_*_9_, *avr_a_*_13_). Log2 expression levels for *Bln1* **(A)**, *Bln2* **(B)**, *Importin α-1b* **(C)**, and *Sec61 γ* **(D)** are plotted in graphs. INOC, inoculation; NON-INOC, Non-inoculated. Error bars represent the standard errors (SE).

To interrogate the GeneChip data, we conducted mixed linear model analyses of the normalized signal intensities for each of the 22,840 Barley1 probe sets (Caldo et al., 2004, see Materials and Methods). Using a stringent threshold *p* < 0.0001 and false discovery rate (FDR) < 5%, 47 genes were suppressed or induced in BSMV:Bln1_248_ silenced plants as compared to the BSMV:00 controls in the HOR11358 (*Mla9*) background. Using the same threshold criteria, 48 genes were similarly affected in the Clansman (*Mla13*) background (Figure 7). Many of the genes affected by silencing *Bln1* in HOR11358, as opposed to Clansman, had dissimilar annotations; this could be due to genotype-specific silencing, genotype-specific probe-set efficiency, or it could reflect the threshold *p*-value we selected (i.e., genes in one background may still be significant, but at a less conservative threshold). Nevertheless, six of these genes were suppressed in common, including the *Bln1* target, represented by Barley1 Contig12219_at [p = 4.66^E-19(HOR11358)^/p = 8.30^E-06(Clansman)^] (Figure 8A; Supplemental Table S1). *Bln2* (Contig26496_at; p = 4.99^E-06^) was suppressed along with *Bln1* in the HOR11358 (*Mla9*) background (Figure 8B), but was not significant at the selected threshold *p* < 0.0001 in the Clansman (*Mla13*) background.

The experiment also yielded many genes that were influenced by infection with BSMV:00, in addition to BSMV:Bln1_248_ (Supplemental Table S1). This may be due to an overlap in general defense gene functions, or may represent strictly *BSMV*-dependent responses. Although one must be cautious regarding overlap in general defense-gene functions, *BSMV* has been shown not to interfere with infection of *Blumeria graminis* f. sp. *tritici*, the causal agent of powdery mildew in wheat (Tufan et al., 2011). However, to specifically understand *Bln1*-regulated targets, we restricted our follow-up functional assays to genes with no confounding effects (Mock vs. BSMV:00 *p* > 0.01).

In addition to the newly identified genes, we also discovered that *Bln2* (Contig26496_at) was suppressed in *Bln1* silenced plants (Figure 8B). Sequence comparison of *Bln1* and *Bln2* indicates that these two genes share significant identity (Figure 4; Meng et al., 2009). Although the *Bln1* silencing construct was designed across a 3′ divergent region, the two *Bln* family members share 13 contiguous nucleotides, indicating that *Bln2* might be unintentionally silenced (Jackson et al., 2003).

### Functional characterization of conserved genes suppressed upon *Bln1* silencing

Analysis of the cohorts described above should provide mechanistic clues to the function of *Bln1* in innate immunity. For example, the most significant candidate from this comparison is Barley1 Contig3615_at [p = 4.85^E-11(HOR11538)^/p = 1.0^E-8(Clansman)^], representing the gene encoding Importin subunit α-1b, which is suppressed in BSMV:Bln1_248_ silenced plants (Figure 8C). Importin subunit α-1b localizes to the perinuclear region of the cytoplasm, where it binds specifically to substrates containing a nuclear localization signal (NLS) and promotes docking of these substrates to the nuclear envelope for subsequent import (Jiang et al., 2001). A homolog of Importin α is also involved in innate immunity in Arabidopsis (Palma et al., 2005). Silencing of *Bln1* also results in the suppression of genes encoding components in the protein secretory pathway, including Sec61 γ, represented by Contig3680_at [p = 2.89^E-05(HOR11538)^/p = 9.31^E-05(Clansman)^] (Figure 8D). Sec61 γ protein is a component of the SEC61 complex that is a conserved protein-conducting channel for secretory protein translocation across the endoplasmic reticulum (ER) membrane (Osborne et al., 2005). Also represented in the common set of six is a gene encoding a putative cysteine protease inhibitor (HV10C01u_s_at). This protein is similar to maize CC9, an apoplastic cysteine protease inhibitor that suppresses host immunity to *Ustilago maydis* (Van Der Linde et al., 2012). Another gene in the conserved set (Contig12608_at) encodes a protein of unknown function but with a classic nuclear localization signal (Dinkel et al., 2014). Genes represented by Contig12219_at (*Bln1*), Contig3680_at (*Sec61 γ*), HS16M03u_x_at (unknown), and HV10C01u_s_at (cysteine protease inhibitor) are induced by *Bgh* infection, whereas, Contig3615_at (*Importin α-1b*) and Contig12608_at (NLS protein) are not. Nevertheless, transcript accumulation of all six is suppressed in *Bln1*-silenced plants (Figure 7).

**Figure 9:**
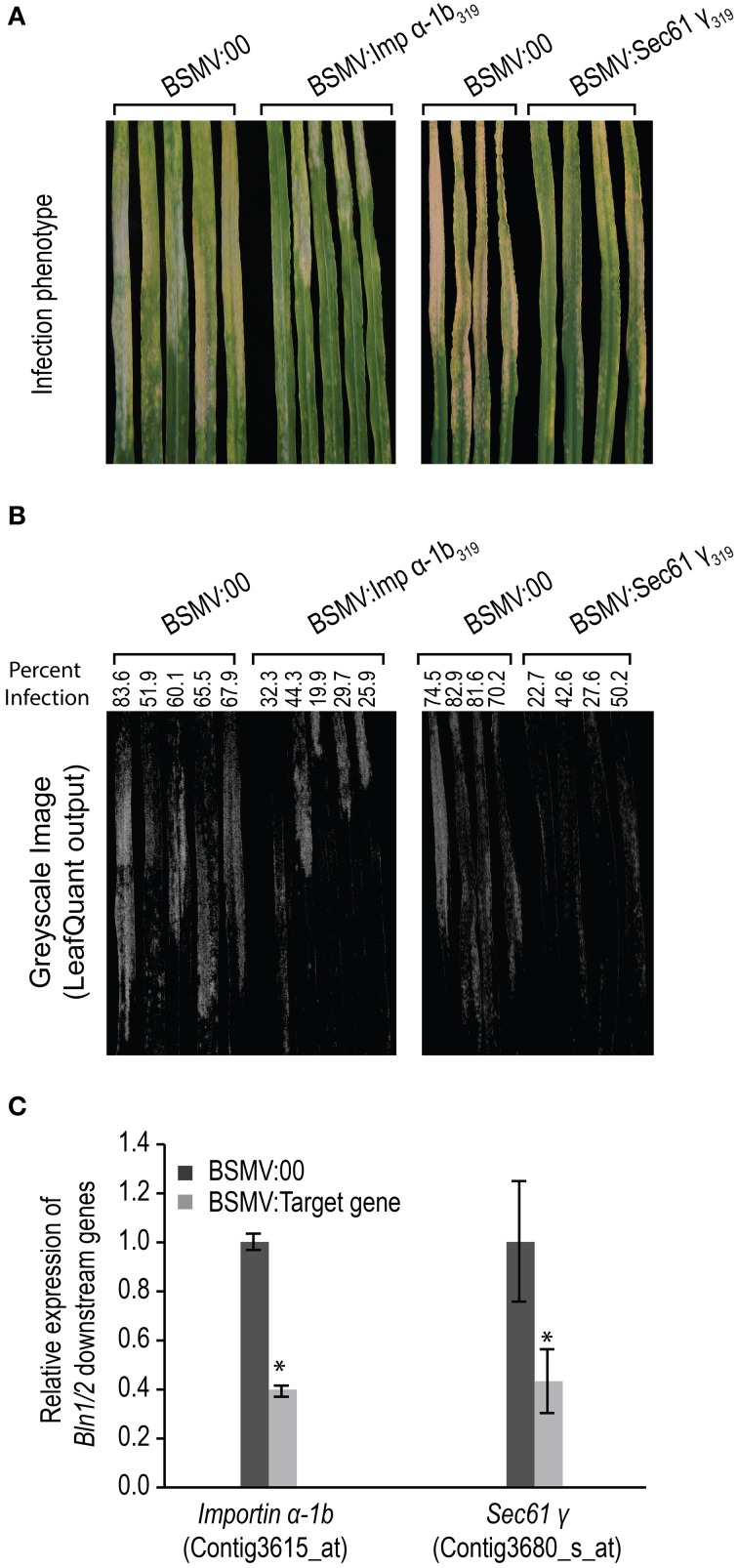
**Infection phenotype of** ***Blumeria graminis*** **f. sp**. ***hordei*** (***Bgh***) **for leaves silenced for** ***Importin α-1b*** **and** ***Sec61*** **γ**. Gene silencing was performed on HOR11358 (*Mla9*) plants and mediated by *Barley stripe mosaic virus* (*BSMV*). Seven-day-old plants were treated with buffer and *BSMV*. Twelve days after buffer and *BSMV* treatments, plants were inoculated with compatible *Bgh* isolate 5874 (*AVR_a_*_1_, *AVR_a_*_6_, *avr_a_*_9_, *avr_a_*_13_). The infection phenotypes were photographed 7 days after inoculation and hyphal growth area for each leaf was quantified by LeafQuant-VIGS software (Table 1). RNA samples for gene expression analysis were prepared from the same quantified leaves and gene levels were analyzed by quantitative reverse-transcriptase polymerase chain reaction analysis on transcript accumulation. The amount of RNA in each reaction was normalized using primers specific for barley Actin. The 2^−ΔCT^ method was used to calculate the target gene expression for each individual silencing construct as compared with BSMV:00 treated plants (Schmittgen and Livak, 2008). Fold change due to silencing is calculated by dividing the expression mean value for the targeted gene in silenced plants by the mean value measured in BSMV:00-treated plants (^*^ designates that *p* < 0.05). Bars represent standard error calculated from at least five independent plants from two replicate experiments. The average value in BSMV:00 samples was set to 1.0. **(A)** Infection phenotype of *Bgh* on *Importin α-1b* and *Sec61 γ* silenced leaves. **(B)** LeafQuant outputs and quantification fungal infection. **(C)** Relative expression of *Importin α-1b* and *Sec61 γ* in *BMSV* induced gene silenced leaves.

Based on these predicted annotations, we then selected a sub-set for functional analysis via *BSMV*-mediated gene silencing. Both *Importin α-1b* (Contig3615_at) and *Sec61 γ* (Contig3680_s_at), were introduced into the BSMV-VIGS system as BSMV:Imp α-1b_319_ and BSMV:Sec61*γ*_319_, respectively, and plants were subjected to silencing as described above. To alleviate off-target silencing, the chosen genes were aligned to the barley genome resource (Mayer et al., 2012). Subsequently, unique, single-copy regions of each target gene were used to design BSMV-VIGS primers (Supplemental Table S2), and each construct was bombarded in at least two independent replicates of 10 plants each. As shown in Figure 9, both of these genes impact powdery mildew development, as demonstrated by significantly less fungal colonies and hyphal growth on the surface of epidermal cells, as compared to BSMV:00 controls (Figures 9A,B, Table 1). qRT-PCR on RNA isolated from the silenced leaves as well as BSMV:00 controls confirmed that transcript accumulation for the target genes was suppressed (Figure 9C). These data suggest that both *Importin α-1b* and *Sec61 γ* play negative roles in barley innate immunity to *Bgh*.

## Discussion

BLUFENSIN1 (BLN1) and BLUFENSIN2 (BLN2), two members in a monocot-specific family of cysteine-rich peptides, are barley susceptibility factors to powdery mildew (Meng et al., 2009). Both BLN1 and BLN2 reveal structural and sequence similarities to knottins, small disulfide-rich proteins characterized by a unique “disulfide through disulfide knot” (Combelles et al., 2008). *Bln* family members are highly upregulated upon fungal infection and BLN proteins can be secreted into the apoplast. Although silencing *Bln* does not break *Mla* [Nucleotide binding, Leucine rich repeat (NLR)]-mediated resistance, knockdown of *Bln1* increases barley innate immune responses and overexpression renders the barley host supersusceptible in compatible interactions.

**Figure 10:**
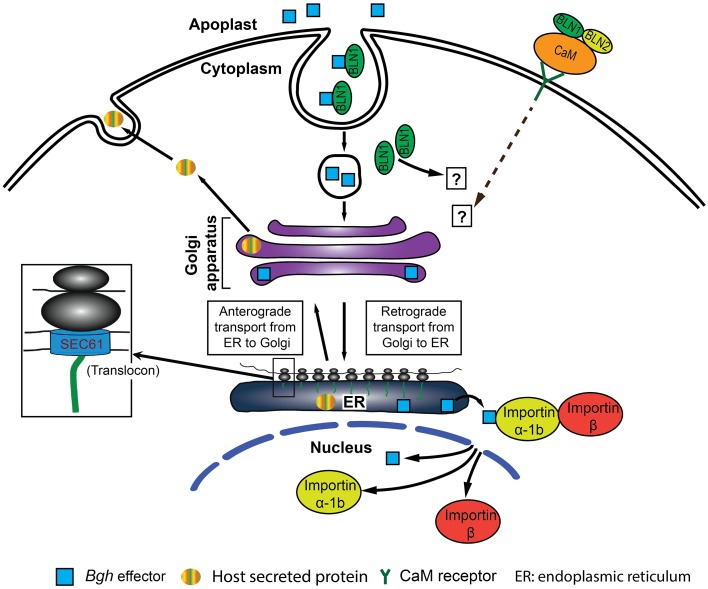
**Predictive model for BLN1 and BLN2 function in the barley immune system**. (1) BLN (green filled circle) can function as a ligand and binds with effectors in the apoplast to promote barley-*Bgh* compatible interactions. (2) The BLN-effector complex then can enter plant cells by endocytic routes, and move to the ER region through a retrograde secretory pathway. (3) Subsequently, effectors could be released into the cytoplasm through the SEC61 pore-forming complex and picked up by Importin α-1b and translocated into the nucleus by the Importin complex to regulate gene expression. (4) The level of BLN1-effector complex may be the positive force regulating the expression of Importin α-1b and Sec61 γ to titrate the BLN-effector level. Alternatively, BLN binds CaM, a Ca^2+^ sensor, in the apoplast and changes CaM conformation to alternate the interaction of CaM with its receptor in the plasma membrane (Cui et al., 2005; Wang et al., 2009) to positively regulate the state-steady levels of Importin α-1b and Sec61 γ. In both cases, BLN, Importin α-1b, and Sec61 γ proteins are positive regulators for barley-*Bgh* compatible interactions (or negative regulators for innate immunity). Silencing of *Bln1* results in the suppression of *Importin α-1b* and *Sec61 γ*, impairing protein anterograde and retrograde trafficking as well as translocation into the nucleus required for barley-*Bgh* compatible interaction.

Based on these observations, we postulate that BLN family members are potential signal molecules co-opted by *Bgh* effectors to bypass innate immune systems and colonize the host. In turn, interactors or partners of BLN would also be expected to play key roles in mediating the plant immune response. This hypothesis is supported by the observed interaction between BLN1 and BLN2, and barley calmodulin (CaM) (Figure 5). CaM, as a universal Ca^2+^ sensor, plays essential roles in regulating numerous intracellular processes, including plant defense (Kim et al., 2002; Reddy et al., 2011; Bender and Snedden, 2013). The BLN protein may function as a ligand to interact with CaM and change its conformation to alter downstream CaM signaling (Figure 10). Alternatively, BLN may interact with barley partner(s) or *Bgh* effector(s) to favor basic compatibility.

### Nuclear import and the secretory pathway—pathogen effector import systems in host plants

Negative regulation of the basal defense pathway prevents unchecked potentiation of the response and deleterious effects on normal cell functions (Ge et al., 2007). Forty-seven and forty-eight genes were identified in HOR11358 (*Mla9*) and Clansman (*Mla13*), respectively, that are significantly differentially expressed (*p* < 0.0001) when *Bln1* is silenced (Figure 7, Supplemental Table S1). Six of these genes were suppressed in common between the two backgrounds. Of these, *Importin α-1b* and *Sec61 γ* are involved in protein trafficking, the CC9 homolog (HV10C01u_s_at) is a putative apoplastic cysteine protease inhibitor, and finally, the gene represented by Contig12608_at, encodes an unknown protein with a classic nuclear localization signal (Dinkel et al., 2014). Silencing two (*Importin α-1b* and *Sec61 γ*) of the conserved set of six genes significantly reduced host susceptibility in compatible interactions of barley and *Bgh* (Figure 9), and CC9 suppresses host immunity to *Ustilago maydis* in maize (Van Der Linde et al., 2012), suggesting that transcription of these plant genes is essential for the fungus to successfully colonize host cells.

It has become evident that the interaction of pathogen effectors and resistance proteins in the nucleus is critical to *R*-gene-mediated resistance (Burch-Smith et al., 2007; Shen et al., 2007; Tameling and Baulcombe, 2007; Wirthmueller et al., 2007; Liu and Coaker, 2008; Tameling et al., 2010). Data presented here indicates that translocation of protein into the nucleus is a key step in innate immunity as well. Importin α in pepper was shown to interact with AvrBs3, a type III-secreted effector from *Xanthomonas campestris* pv. *vesicatoria*, both in yeast and *in vitro* through a nuclear localization signal (NLS) (Szurek et al., 2001). Given that the gene encoding Importin subunit α-1b (Contig3615_at) is significantly down-regulated in *Bln1*-silenced plants, we postulate that a function of BLN1, even in the absence of *Bgh* infection (Figure 8C), could be to sustain transcript accumulation of Importin. Induction of BLN1 by *Bgh* may enhance the translocation of select effectors from the apoplast to the nucleus, which might be necessary for the fungus to colonize its barley host.

Such a scenario, though not reported before in cereal-fungal interactions, is not without precedent. In the interaction of Arabidopsis with *Agrobacterium*, a historically important pathogen most widely know for its role in plant transformation (McCullen and Binns, 2006), multiple Importin α proteins interact with both *Agrobacterium* VirD2 and VirE2. However, Importin α-4 appears to be the most crucial isoform for transfer of Vir proteins to the plant cell nucleus (Bhattacharjee et al., 2008).

It should also be noted that although two full-length Importin α isoforms have been identified in barley (Importin α-1a and Importin α-1b, represented by probe sets Contig4129_at and Contig3615_at, respectively), only expression of Importin α-1b is significantly affected by *Bln1* silencing. This indicates that Importin α-1b is specifically involved in BLN-mediated resistance to barley powdery mildew.

Nonetheless, Importin α-1b in barley and Importin α3 in Arabidopsis (Palma et al., 2005) have opposite effects on plant defense against pathogens. This suggests that different isoforms of Importin α have substrate-specific recognition to differentially regulate plant immunity. Substrate-specific recognition of Importin has also been observed for nuclear import of proteins involved in rice photomorphogenesis (Jiang et al., 2001) and for neural differentiation of mouse embryonic stem cells (Goldfarb et al., 2004; Yasuhara et al., 2007, 2013), signifying the diverse temporal and spatial regulation influenced by Importin isoforms in plant and animal systems.

Silencing of *Bln1* also results in the coordinate suppression of genes encoding components in the protein secretory pathway, including the SEC61 complex. The SEC61 complex is a conserved protein-conducting channel for translocation across the endoplasmic reticulum (ER) membrane (Osborne et al., 2005; Kelkar and Dobberstein, 2009; Park and Rapoport, 2012) and is required for induction of systemic aquired resistance to *Pseudomonas syringae* pv. *maculicola* in Arabidopsis (Wang et al., 2005). Recently, the Sec61 β subunit in barley has been shown to be an ER protein transporting pore that is required for host susceptibility to powdery mildew (Zhang et al., 2013).

### Does BLN1 drive protein trafficking?

The protein secretory pathway starts with insertion of protein into the SEC61 translocon complex and involves a series of steps by which proteins are transported between organelles in anterograde or retrograde directions. Protein trafficking into the nucleus includes the interaction of transported targets with the Importin complex. The secretory pathway plays a vital role in plant disease resistance (Kwon et al., 2008a,b; Rojo and Denecke, 2008; Wang and Dong, 2011). Down-regulation of multiple components in the secretory pathway in *Bln1* silenced plants point to a role of *Bln1* in the regulation of this pathway. One possibility is that BLN1 may interact with host-secreted proteins required for pathogen-host interaction. Silencing of *Bln1* may reduce the amount of these BLN1 interacting host-secreted proteins, resulting in down-regulation of anterograde protein transport. Alternatively, interaction of BLN1 with *Bgh* or host secreted proteins could signal the entry of BLN interactors into the host cell via the retrograde pathway (Spooner et al., 2006; Johannes and Popoff, 2008; Dong et al., 2013; Drerup and Nechiporuk, 2013; Koyuncu et al., 2013). Silencing of *Bln1* reduces these interactions and therefore release the demand for components involved in protein trafficking pathway, such as Sec61 γ and Importin α-1b. A third scenario may involve BLN1 interacting with a specific signal molecule, such as the Ca^2+^ sensor CaM, to positively regulate transcript accumulation of genes encoding components involved in protein trafficking, such as *Sec61 γ and Importin α-1b*. Knock down of *Bln1* would suppress the expression of genes encoding these components (Figure 10). All three hypotheses are supported by the observation that *Bln* family members regulate the expression of genes implicated in protein trafficking, overexpression of *Bln* renders barley more susceptible to *Bgh* (Figure 2), and that silencing of *Bln1, Importin α-1b*, and *Sec61 γ* increases barley innate immunity (Figures 6, 9). Thus, BLN proteins may modulate protein transport in barley-*Bgh* interactions and BLN levels influence protein trafficking in infected barley cells.

### Summary: are BLN1 and BLN2-potential host-targeting signals for *Bgh*?

In the interaction between barley and *Bgh*, transfer of signals may be expected to occur between host and pathogen during formation of the perihaustorial interface (Dodds et al., 2004, 2006).

From an evolutionary standpoint, genes in redundant networks with an incredible level of buffering capacity imply minimal selective pressures acting on these genes. One example was discussed by Xu et al. (2006), where *Atwrky18*/*Atwrky40* and *Atwrky18*/*Atwrky60* double mutants were more resistant to *Pseudomonas syringae* DC3000 but more susceptible to *Botrytis cinerea* infection, but single *Atwrky* mutants behaved similar to wild-type plants. BLN1 and BLN2 are potentially part of a redundant set of negative regulators for plant defense.

Both BLN1 and BLN2 are highly induced upon *Bgh* inoculation and transient overexpression increases barley plants susceptibility; these results are consistent with the silencing results. We further identified that BLN proteins could be secreted into the apoplast and interact with each other and CaM in plant cells. Thus, BLN could act as potential host-targeting signals for *Bgh* to colonize in plant cells; the interactions between BLN and *Bgh* effectors could fine-tune sets of protein pathways, which might be involved in transporting fungal proteins into the host cells.

## Materials and methods

### Plant materials and fungal isolates

Seedlings of barley lines Black Hulless, CI 16151 (*Mla6*), HOR11358 (*Mla9*), and Clansman (*Mla13*) were used for functional analysis. Virus infected barley was maintained in a growth chamber with a 16 h photoperiod with light intensity at 550 μmol m^−2^ s^−1^ and a daytime temperature of 24°C and dark temperature of 20°C. *Bgh* isolate 5874 (*AVR_a_*_1_, *AVR_a_*_6_, *avr_a_*_9_, *avr_a_*_13_) was propagated on *H. vulgare* cv. Manchuria (CI 2330) in a controlled growth chamber at 18°C (16 h light/8 h darkness).

### BLN1 and BLN2 subcellular localization

Total RNA was extracted from CI 16151 (*Mla6*) plants 20 hai with *Bgh* isolate 5874 (*AVR_a_*_6_) according to the method of Caldo et al. (2004). First-strand cDNA was synthesized using 2 μg of total RNA, oligo(dT)_20_ primer and Superscript reverse transcriptase III (Invitrogen, Carlsbad, CA). Subsequently, first strand cDNA was used as the template to amplify *Bln1* and *Bln2* coding sequences with/without signal peptides or the signal peptide regions; Primers were designed according to *Bln1* EST sequence (GeneBank Accession no. is FJ156737) and *Bln2* EST sequence (GeneBank Accession no. is FJ156745) and listed in Supplemental Table S2.

BLN-GFP chimeric constructs were made using overlapping PCR. First, full length *Bln1* was amplified using primer pair Bln1-NcoN_pf1 and Bln1-C_pr1; *GFP* was amplified with pEGFP (Clontech Laboratories, Inc., Mountain View, CA) as template and primer pair GFP-FKS_pf1 and GFP-Bam_pr1. The final PCR was performed using PCR products of the previous two reactions as template and primer pair Bln1-NcoN_pf1 and GFP-Bam_pr1. The final PCR product was digested with *Nco*I and *Bam*HI and ligated into similarly treated pTRL2 (Restrepo et al., 1990) to yield p35S:BLN1+SP, harboring coding regions for full-length BLN1 and GFP. A similar strategy was adopted to make the *Bln1* signal peptide-GFP construct, p35S:BLN1_SP only, as well as *Bln1*-GFP construct absent the *Bln1* signal peptide, p35S:BLN1-SP. When making corresponding Bln2-GFP constructs (p35S:BLN2+SP, p35S:BLN2_SP only and p35S:BLN2-SP), PCR products were digested with *Nco*I and *Sma*I and inserted into similarly treated pBLN1+SP.

These constructs were delivered into onion epidermal cells by using biolistic PDS-1000/he system (Bio-Rad, Hercules, CA, USA) as described by Elling et al. (2007). After bombardment, epidermal peels were incubated for 24 h in the dark at 20–25°C. Plasmolysis of onion epidermal cells was attained by soaking the peels in 1 M sucrose solution for 20 min. A Zeiss Axio Imager M.1 microscope (Zeiss, Inc., Thornwood, NY) was used for observation. At least three independent replicate experiments were conducted.

To visualize GFP in the apoplast, onion epidermal cells were incubated in agar medium supplemented with 3% sucrose (Murashige and Skoog, 1962) for 20 h, and transferred to 20 mM Pipes-KOH (pH 7.0) for 4 h on half-concentrated Murashige and Skoog (1962) agar medium supplemented with 1% sucrose (Genovesi et al., 2008). The pH 7.0 medium neutralizes the normally acidic apoplast in order to observe the fluorescence patterns.

### Overexpression of *Bln1* and *Bln2* by using the *BSMV* system

To introduce a foot- and-mouth disease virus (FMDV)-2A self-cleavage peptide and GFP for expression of foreign gene, pBPMV-IA-V5 (Zhang et al., 2010) was used as template with primer pair BS3-G4F1 and BS3-G4R1 for PCR to produce DNA fragment A, which was used as template with primer pair BS3-G4F2 and BS3-G4R2 to produce DNA fragment B. BSMV:γ (Meng et al., 2009) was used as template with primer pair BSMV-R3-F3 and BS3-4Rev for PCR to produce DNA fragment C. DNA fragments B and C were then used for overlapping PCR with primer pair BSMV-R3-F3 and BS3-G4R2 for PCR to produce DNA fragment D. Product D was digested with *Bgl*II and *Kpn*I and ligated into BSMV: γ digested by *Bgl*II and *Kpn*I to produce pBSMV-OEx:GFP.

To make *Bln1* and *Bln2* overexpression constructs, *Bln1* and *Bln2* cDNA described above were used as templates and primer pairs BSBln1Ov_pf1/BSBln1Ov_pr1 or BSBln2Ov_pf1/BSBln2Ov_pr1 were used respectively (Supplemental Table S2). PCR fragments contained an introduced *Stu*I and *Bam*H1 recognition sites at the 5′ and 3′ ends, respectively and were inserted into the *Stu*I and *Bam*H1 sites of pBSMV-OEx:GFP, the resulting vectors were designated as pBSMV-OEx:Bln1 and pBSMV-OEx:Bln2, respectively.

DNA bombardment and subsequent virion mechanical infection on HOR11358 (*Mla9*) plants was performed according to Meng et al. (2009). A Zeiss Axio Imager M.1 microscope (Zeiss, Inc., Thornwood, NY) was used for observation the GFP. At least two independent replicate experiments were performed.

### BLN1 and BLN2 interactions via bimolecular fluorescence complementation (BiFC)

Site-directed mutagenesis was carried out on *Bln1* and *Bln2* full length ORFs using QuickChange™ site-directed mutagenesis kit (Stratagene, La Jolla, CA). Primers used for amplification and mutagenesis are listed in Supplemental Table S2. From the BLN1 start codon, amino acid residues 30 and 42 were changed from Q to G and residues 36 and 45 were changed from C to G. For BLN2, amino acid residues 30 and 44 were changed from Q to G and residues 37 and 47 were changed from C to G. The resulting products contained *Eco*RI and *Bam*HI restriction sites respectively, and cloned into *Eco*RI-*Bam*HI sites of pSAT4-nEYFP-C1 or pSAT4-cEYFP-C1 to generate pSAT4-nEYFP-Bln1, pSAT4-nEYFP-Bln2, pSAT4-cEYFP-Bln1, and pSAT4-cEYFP-Bln2, respectively. For coexpression, particle bombardment was performed using onion epidermal cells. Gold particles (1.6 μm diameter) (Bio-Rad) were washed with 100% ethanol and coated with 1.25 μg of each DNA using standard procedures. cDNA-coated gold particles were bombarded at 1100 p.s.i. and 9 cm distance using a Biolistic Particle Delivery System PDS-1000/He (Bio-Rad). Bombarded tissues were incubated at 25°C in darkness for ~24 h before being assayed for YFP activity. The bright-field and fluorescent images were taken using the Zeiss Axiovert 100 microscope with appropriate YFP filter.

### BLN interactions with calmodulin via bimolecular fluorescence complementation (BiFC)

Calmodulin full length ORFs were PCR-amplified using primers listed in Supplemental Table S2. The resulting product containing *Eco*RI and *Bam*HI restriction sites was cloned into *Eco*RI-*Bam*HI sites of pSAT4-cEYFP-C1 to generate pSAT4-cEYFP-CaM. This was combined with Bln1 or Bln2 pSAT4 constructs as described above for bombardment and microscopic observation. At least three independent replicate experiments were conducted.

### Statistical analysis of YFP cell count data

All cells exhibiting YFP were counted for each of the 3 independent biological replications. A mixed linear model analysis of observed YFP cell count data was conducted using PROC MIXED in SAS software. The model used total YFP cell count as the response and included random effect for replications. Four contrasts in SAS software were used to compare different controls (*Bln1* and *CaM, Bln2* and *CaM, Bln1* and *Bln2, Bln2* and *Bln1*) with their respective treatments. The least square means were correlated and when the *p*-values were adjusted using the Dunnett (1980) method, PROC MIXED used the factor-analytic covariance approximation described in Hsu (1992).

### Target synthesis and GeneChip hybridization

Total RNA was isolated using a hot (60°C) phenol/guanidine thiocyanate method described by Caldo et al. (2004). Trizol-like reagent was made from 38% saturated phenol (pH 4.3), 0.8M guanidine thiocyanate, 0.4M ammonium thiocyanate, 0.1M sodium acetate (pH 5.0) and 5% glycerol (Fisher Scientific, Pittsburg, PA). RNA was purified further using RNeasy columns (Qiagen, Valencia, CA). Probe synthesis, labeling, and hybridization to Barley1 GeneChip probe arrays (Affymetrix #900515; Close et al., 2004) were performed using One Cycle and GeneChip IVT labeling protocols based on the Affymetrix manual (Affymetrix, Santa Clara, CA) at the Iowa State University GeneChip Core facility.

### Normalization and mixed linear model analysis of Barley1 genechip data

As described previously (Caldo et al., 2004; Wise et al., 2007), we conducted mixed linear model analyses of the normalized signal intensities for each of the 22,840 Barley1 probe sets (Wolfinger et al., 2001). RMA normalization and data transformation was done using package affy in BioConductor/R. Mixed linear model analysis was conducted using PROC MIXED in SAS software. The model used RMA normalized expression values as the response, replication, inoculation and treatment (genotype^*^vector) as fixed factors, and replication^*^inoculation and replication^*^treatment as random factors. Contrasts in SAS software (SAS Institute Inc., Cary, NC, U.S.A.) were used to compare transcript levels between treatments (Mock vs. BSMV:00; BSMV:00 vs. BSMV:Bln1_248_) of a specific genotype [HOR11538 (*Mla9)* or Clansman (*Mla13*)] after infection with *Bgh* 5874. *Q*-values were estimated using the smoother method described in Storey and Tibshirani (2003). From these analyses, we expected to identify sets of genes involved in *Bln1*-mediated compatibility or incompatibility, and also genes that are perturbed in response to *BSMV* infection, in both *Bgh* inoculated vs. non-inoculated reference plants.

### BSMV-VIGS

Inserts for BSMV-VIGS were amplified by PCR using primers that add *Pac*I and *Not*I restriction sites to the 5′ end and 3′ end, respectively (Supplemental Table S2). These sites enable ligation of the fragment in antisense orientation into the BSMV:γ vector. Silencing experiments were performed as described previously (Meng et al., 2009; Xi et al., 2009; Meng and Wise, 2012; Xu et al., 2014). Plants were maintained for 12 days in a growth chamber (Percival Scientific, Perry, IA, USA) with 16 h of light at 24°C (550 μmol m^−2^ s^−1^) and 8 h darkness at 20°C. Plants were then inoculated with *Bgh* isolate 5874 (*avr_a_*_9_) conidiospores [compatible interaction with HOR 11358 (*Mla9*)] and maintained in a growth chamber 16 h of light/ 8 h of darkness at 18°C. The infection phenotype was monitored for 7 days.

### Quantitative real-time PCR

Barley leaves were pulverized in liquid nitrogen and total RNA extracted using Trizol-like reagent (Caldo et al., 2004). Genomic DNA was degraded by RNase-free DNase I (Ambion, Austin, TX, U.S.A.). SuperScript III reverse transcriptase (Invitrogen, Carlsbad, CA, USA) was used to synthesize first strand cDNA using 2 μg total RNA and oligo(dT)_20_ primer. This cDNA was used as a template for qRT-PCR to determine expression of various target genes to barley Actin. The qRT-PCR was performed using a Bio-Rad iCycler (Bio-Rad, Hercules, CA, USA). Conditions for 20 μL reactions using PerfeCTa® SYBR® Green FastMix® for iQ (Quanta Biosciences, Gaithersburg, MD) were 95°C for 3 min, followed by 40 cycles of 95°C for 15 sec and 60°C for 1 min, then a melt curve was determined by starting at 55°C for 10 s and then increasing by 0.5°C every 10 s for 80 cycles. Three technical replicates for each biological sample in addition to four or five biological samples per treatment were included in each experiment. Target gene expression was calculated using the 2^−ΔCT^ method for the BSMV:target gene and BSMV:00-treated plants. The fold change due to silencing was calculated by dividing the expression value for each BSMV:target gene treated leaves by the mean value measured in BSMV:00 treated plants (Schmittgen and Livak, 2008).

### Imaging of *Bgh* infection phenotypes

At seven days after powdery mildew inoculation, third leaves of *BSMV* treated plants were randomly selected and photographed at high resolution (2592 pixels × 3456 pixels, i.e., 9 Megapixel at 4:3 aspect ratio) using a Canon PowerShot SX110 IS and the Vidpro professional Photo and Video LED light kit model Z-96K. The leaves were set on black felt for uniform, high contrast background. Subsequent images were analyzed using an in-house pattern-recognition software, designated LeafQuant-VIGS, developed using MathWorks® MATLAB® 7.14 and Image Processing Toolbox™ 8.0. Starting with high-resolution RGB images of each leaf, LeafQuant-VIGS first defines the edges, then detects the background and converts it to uniformly true black, converts the high-resolution color RGB image to an 8-bit gray-scale image with 256 shades of gray, and outputs histograms of the hyphal distribution per leaf, which then reports mean, median, and quantiles of the results as a csv (comma separated values) file for further processing (Whigham et al., 2015). Because elongating secondary hyphae (ESH), an indicator of functional haustoria (Ellingboe, 1972), are white and the barley leaf is green, these differences can be used to quantify fungal growth in terms of percent infection. LeafQuant-VIGS software is offered under MIT license via Github at http://git.io/leafquant.

### Analysis of LeafQuant-VIGS data

A separate linear model analysis of the LeafQuant-VIGS data was conducted for each silencing and over-expression construct using the package MULTCOMP in R programming language (Hothorn et al., 2008). All models used percent infection as the response. The model was used to compare the mean percent infection of BSMV:00 treated leaves to the mean percent infection for mock and BSMV:construct treated leaves. *P*-values were adjusted for multiple testing using the Dunnett method (Dunnett, 1955).

### Data access

All detailed data and data from expression profiling have been deposited as Accession number BB101 in PLEXdb (http://plexdb.org/) (Dash et al., 2012). Files can be downloaded as batch files in MAGE-ML, CSV, CEL, DAT, or expression data formats at the Download Center or downloaded as individual CEL, CHP, DAT, or EXP files under “browse experiments.” Data has also been deposited as Accession number GSE61644 at NCBI-GEO.

## Author contributions

Conceived and designed the experiments: YM, WX, DN, and RW. Performed the experiments: YM, WX, GF, and RW. Analyzed the data: WX, YM, PS, GF, DN, and RW. Contributed reagents/materials/ analysis tools: YM, WX, PS, GF, DN, and RW. Wrote and edited the paper: WX, YM, PS, GF, and RW.

### Conflict of interest statement

The authors declare that the research was conducted in the absence of any commercial or financial relationships that could be construed as a potential conflict of interest.
