# Supplementary material for: The knottin-like Blufensin family regulates genes involved in nuclear import and the secretory pathway in barley-powdery mildew interactions
Source: Front Plant Sci. 2015 Jun 4;6:409. doi: 10.3389/fpls.2015.00409 (PMC4454880; doi:10.3389/fpls.2015.00409)
Supplement: Supplementary file 2 [file Table2.DOCX]

**Supplemental Table S2 (Xu et al. 2015).** Primers for making constructs of gene silencing, qRT-PCR gene,

subcellular localization (SL), gene overexpression (OEx), BiFC and site-directed mutagenesis (Mutation).

| Primer Name | Primer Sequence (5’→ 3’) | Function |
| --- | --- | --- |
| BSMV:3615_pf1 | ^a^ATATTAATTAACCAAAGTGGTTGTTGAGTTCGG | Silencing |
| BSMV:3615_pr1 | ^a^TATGCGGCCGCCTCCTCGTCCTGAGAGTGGATAAG | Silencing |
| BSMV:3680_pf2 | ^a^TATTAATTAATCGAAAGGAGTTTACCAAGGTGG | Silencing |
| BSMV:3680_pr1/2 | ^a^TATGCGGCCGCCGGTATCAGGTTCAAAACTGCTCC | Silencing |
| BSMV:Bln1_pf1 | ^a^ATATTAATTAAACGAGGATATGGCAAAGAACTAC | Silencing |
| BSMV:Bln1_pr1 | ^a^TATGCGGCCGCTAATAGTAGAGCAGCTTCCAACG | Silencing |
| 3615q_pf1 | GGTCAGTCTTCAAGGTCAACCG | qRT-PCR |
| 3615q_pr1 | AACCCAACTCTCCCGACAACAC | qRT-PCR |
| 3680q_pf1 | GGTGTTCATTCATGGAGGCG | qRT-PCR |
| 3680q_pr1 | CGGTATCAGGTTCAAAACTGCTCC | qRT-PCR |
| BSMV:Bln1qRT_pf1 | CGAGATGGACCATTTCTGTGCA | qRT-PCR |
| BSMV:Bln1qRT_pr1 | TACCAGGTCACAGTACAAACA | qRT-PCR |
| BSMV:Bln2qRT_pf1 | GATGACTCTGGTCCGATTCAGC | qRT-PCR |
| BSMV:Bln2qRT_pr1 | CCAATGCGAGATCCTACCAAATC | qRT-PCR |
| BSBln1Ovt_pf1 | CAAAGAACTACTCCTCTGCGA | qRT-PCR |
| BSBln1Ovt_pr1 | TGAGCCACCATTAGGGATCG | qRT-PCR |
| BSBln2Ovt_pf1 | GCAAACAAGTACTCGGCCAG | qRT-PCR |
| BSBln2Ovt_pr1 | AGCTCTGCTGAATCGGACCA | qRT-PCR |
| BSBln1Ov_pf1 | tacaggcctATGGCAAAGAACTACTCCTCT | OEx |
| BSBln1Ov_pr1 | ttaggatccTGAGCCACCATTAGGGATCGA | OEx |
| BSBln2Ov_pf1 | tacaggcctATGATGGCAAACAAGTACTCG | OEx |
| BSBln2Ov_pr1 | ttaggatccTGAGCCACCACGGATTGGCG | OEx |
| BSMV-R3-F3 | GCACTCACGCAAAGTAAAGGT | OEx |
| BS3-G4F1 | GTATTTACCTTCGCAGGCCTATGAGTAAAGGAGAAGAACTTT | OEx |
| BS3-G4F2 | CCTGATGTTTAAATCTACTGTATTTACCTTCGCAGGCCT | OEx |
| BS3-G4R1 | CAAGAGAAAGTAGCCATCATGGGCCCAGGGTTGGACT | OEx |
| BS3-G4R2 | GTTAAGGTACCACAACACACACAAGAGAAAGTAGCCATCAT | OEx |
| BS3-4Rev | GAAGGTAAATACAGTAGATTTAAACATCA | OEx |
| Bln1-NcoN_pf1 | CATGccatggcaaagaactactcctctgcga | 35S:BLN1+SP |
| Bln1-C_pr1 | ctcaccacccgggtacctgagccaccattagggatcga | 35S:BLN1+SP |
| Bln1-M_pr1 | ctcaccacccgggtacctccaaggatgccaccaacga | 35S:BLN1 SP only |
| Bln1-NcoM_pf1 | CATGCCATGGCACAAGGTGGCCCGTCCA | 35S:BLN1 - SP |
| Bln1-M_pr1 | ctcaccacccgggtacctccaaggatgccaccaacga | 35S:BLN1 - SP |
| Bln2-NcoN_pf1 | CATGCCATGGTAATGGCAAACAAGTACTCG | 35S:BLN2+SP |
| Bln2-C_pr1 | accacccgggtTGAGCCACCACGGATTG | 35S:BLN2+SP |
| Bln2-M_pr1 | ACCACCCGGGTCCCGAGGATGGTACCGA | 35S:BLN2 SP only |
| Bln2-NcoM_pf1 | CATGccatggcaCAACGAGGTGGACCAT | 35S:BLN2 - SP |
| Bln2-M_pr1 | ACCACCCGGGTCCCGAGGATGGTACCGA | 35S:BLN2 - SP |
| GFP-FKS_pf1 | ggtacccgggtggtgagcaagggcgagga | SL-GFP |
| GFP-Bam_pr1 | GCGggatccttacttgtacagctcgtccatg | SL-GFP |
| pSAT4-nEYFP-Bln1_pf1 | cttagaattcTATGGCAAAGAACTACTCCTCT | BiFC |
| pSAT4-nEYFP-Bln1_pr1 | cttaggatccTTATGAGCCACCATTAGGGATCGA | BiFC |
| pSAT4-nEYFP-Bln2_pf1 | cttagaattcTATGATGGCAAACAAGTACTCG | BiFC |
| pSAT4-nEYFP- Bln2_pr1 | ttaggatccTTATGAGCCACCACGGATTGGCG | BiFC |
| Bln1_Q30G_pf1 | ^b^CATCCTTGGAGGAGGTGGCCCGTCC | Mutation |
| Bln1_Q30G_pr1 | GGACGGGCCACCTCCTCCAAGGATG | Mutation |
| Bln1_Q42G_pf1 | ^b^AACCCTGCGGCTGGGCGAAACTGCCC | Mutation |
| Bln1_Q42G_pr1 | GGGCAGTTTCGCCCAGCCGCAGGGTT | Mutation |
| Bln2_Q30G_pf1 | ^b^ACCATCCTCGGGGGACGAGGTGGAC | Mutation |
| Bln2_Q30G_pr1 | GTCCACCTCGTCCCCCGAGGATGGT | Mutation |
| Bln2_Q44G_pf1 | ^b^CTGGTCCGATTGGGCAGAGCTGCCC | Mutation |
| Bln2_Q44G_pr1 | GGGCAGCTCTGCCCAATCGGACCAG | Mutation |
| Bln1_C36G_pf1 | ^b^CCCGTCCAAAGGCAGCAACCCTGCG | Mutation |
| Bln1_C36G_pr1 | CGCAGGGTTGCTGCCTTTGGACGGG | Mutation |
| Bln1_C45G_pf1 | ^b^GCTCAGCGAAACGGCCCGTCGATCC | Mutation |
| Bln1_C45G_pr1 | GGATCGACGGGCCGTTTCGCTGAGC | Mutation |
| Bln2_C37G_pf1 | ^b^ACCATCCACCGGCGATGACTCTGGTC | Mutation |
| Bln2_C37G_pr1 | GACCAGAGTCATCGCCGGTGGATGGT | Mutation |
| Bln2_C47G_pf1 | ^b^TTCAGCAGAGCGGCCCGCCAATCCG | Mutation |
| Bln2_C47G_pr1 | CGGATTGGCGGGCCGCTCTGCTGAA | Mutation |

^a^ BSMV-VIGS inserts were amplified by PCR using primers that add *Pac*I and *Not*I restriction sites (shown in grey)

to the 5’ end and 3’ end, respectively.

^b^ Red nucleotides signify the mutational events encoded in forward primers for site-directed mutagenesis.
